# Case Report: Exome Sequencing Reveals LRBA Deficiency in a Patient With End-Stage Renal Disease

**DOI:** 10.3389/fped.2020.00042

**Published:** 2020-03-11

**Authors:** Christina Taylan, Andrea Wenzel, Florian Erger, Heike Göbel, Lutz T. Weber, Bodo B. Beck

**Affiliations:** ^1^Department of Pediatrics, Faculty of Medicine and University Hospital Cologne, University of Cologne, Cologne, Germany; ^2^Faculty of Medicine and University Hospital Cologne, Institute of Human Genetics, University of Cologne, Cologne, Germany; ^3^Center for Molecular Medicine, Faculty of Medicine and University Hospital Cologne, University of Cologne, Cologne, Germany; ^4^Department of Pathology, Faculty of Medicine and University Hospital Cologne, University of Cologne, Cologne, Germany

**Keywords:** lipopolysaccharide-responsive beige-like anchor protein (LRBA), renal failure, kidney transplantation, tubulointerstitial kidney disease, whole-exome sequencing

## Abstract

**Background:** Lipopolysaccharide-responsive and beige-like anchor protein (LRBA) deficiency is characterized by autoimmunity, chronic diarrhea, and immunodeficiency. Minor renal manifestations have been found in a few patients, but kidney disease has not been systematically studied and may remain underdiagnosed in this highly variable entity.

**Results:** Our patient initially presented with pancytopenia, enteropathy, hypogammaglobulinemia, and failure to thrive at the age of 15 months. Chronic kidney disease was diagnosed at 6 years. A renal biopsy taken at 11 years of age showed interstitial nephritis. The patient progressed rapidly to end-stage renal disease (ESRD) and underwent kidney transplantation at the age of 12 years. Bronchiolitis obliterans, post-transplant lymphoproliferative disease (PTLD), and chronic rejection complicated the post-transplant management. Graft loss required reinstitution of hemodialysis within 3 years. After negative results of different targeted sequencing strategies, exome sequencing identified a homozygous nonsense mutation (p.Q1010^*^) in the *LRBA* gene more than 21 years after the patient's initial presentation.

**Conclusions:** We report here the development of ESRD and long-term follow-up in a patient with LRBA deficiency. A molecular diagnosis in rare (kidney) disease like LRBA deficiency bears many advantages over a descriptive diagnosis. A precise diagnosis may result in improved (symptomatic) treatment and allows differentiating treatment- and procedure-related complications from manifestations of the primary disease.

## Introduction

Many children requiring dialysis and renal transplantation suffer from rare, mostly inherited disorders. More than 400 different rare kidney diseases are currently known (1). New technologies of massive parallel sequencing and improvements in bioinformatics have allowed for a remarkable progress in understanding the Mendelian basis of rare diseases within the past years (1, 2). Lipopolysaccharide (LPS)-responsive beige-like anchor protein (LRBA) deficiency was identified as the cause of common variable immunodeficiency (CVID-8; OMIM #614700) in association with autoimmunity and/or inflammatory bowel disease (IBD)-like phenotype in 2012 (3). LRBA is ubiquitously expressed (3) and involved in the regulation of endosomal trafficking, particularly endocytosis of ligand-activated receptors. LRBA regulates cytotoxic T lymphocyte-associated protein 4 (CTLA-4) expression on the posttranslational level. CTLA-4 constitutes a potent suppressive receptor and immune checkpoint (3). Affected individuals suffer from early childhood onset of recurrent infections, particularly respiratory infections and failure to thrive, and also develop various autoimmune disorders, including idiopathic thrombocytopenic purpura, autoimmune hemolytic anemia, and a wide range of gastrointestinal symptoms including chronic diarrhea. Also, interstitial lung disease (ILD) has been reported in association with LRBA deficiency. The phenotypic spectrum of LRBA deficiency is (inter- and intrafamilial) diverse. Even in cases without overt immunodeficiency, testing for LRBA deficiency should be considered. Immunologic findings may include decreased B cells, hypogammaglobulinemia, and deficiency of CD4^+^ regulatory T cells (Tregs) (4). Besides bone marrow transplantation, potential therapeutic options in these patients include the inhibition of lysosome degradation with chloroquine (to prevent CTLA-4 loss) or treatment with Abatacept (a fusion protein of the extracellular CTLA-4 domain linked to the Fc fragment of human IgG1).

We identified a novel homozygous *LRBA* stop mutation (p.Q1010^*^) in our patient who initially suffered from progressive tubulointerstitial kidney disease and multiple therapy-related complications after kidney transplantation.

## Materials and Methods

### Genetic Analysis

**Figure 1:**
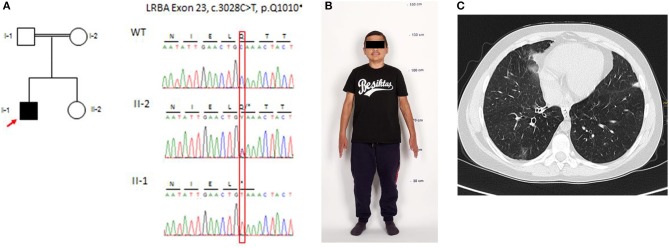
Genetic analysis, physical features (as an adult), and computed tomography (CT) of the chest at the age of 14 years. **(A)** Pedigree of the family: electropherograms depict the relevant sequence section around the causative C-to-T transition at position c.3028 in exon 23 and the generation of a premature stop codon at position p.Q1010*. **(B)** Physical features and final height (133 cm) of the patient (reproduction with permission of the patient). **(C)** Chest CT demonstrates bronchiolitis obliterans, lower lobe right side: small area of ground-glass opaque consolidation, swelling, and widening of bronchial tubes.

Karyotyping, chromosomal microarray, and gene panel (3,613 genes; TruSight One) analysis did not provide a clear genetic yield. Next, we performed whole-exome sequencing (WES) using the Agilent SureSelect Human All Exom V6 enrichment (Agilent Technologies Inc., Santa Clara) followed by next-generation sequencing on an Illumina HighSeq 4000™ sequencing platform (lllumina, San Diego). For whole-exome sequencing filtering and variant calling was performed using the Cologne Center for Genomics' VARBANK database and analysis tool^1^. We filtered for high-quality (coverage >15x; Phred quality score > 25) homozygous variants (>75% allele frequency) with a minor allele frequency (MAF) ≤ 0.01. To exclude pipeline-related artifacts (MAF ≤ 0.01), we filtered against variants from in-house WES datasets from 511 epilepsy patients. Applying these filter criteria yielded only a single homozygous variant associated with Mendelian disease in the LRBA gene [c.3028C>T (p.Q1010^*^)] (Figure 1A). Further comprehensive WES analysis did not yield any additional (likely) pathogenic variants associated with monogenic nephropathy or autoimmunity.

### Immunohistochemistry

**Figure 2:**
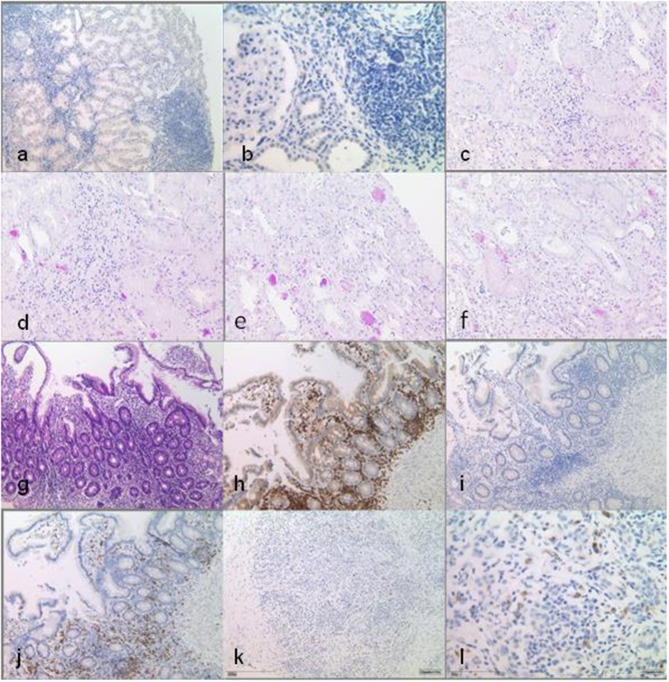
Histopathological findings in multiple biopsy specimen. Native kidney biopsy taken before the start of any immunosuppression shows interstitial infiltration of the lymphohistiocytic cells, with CD80 positivity only in single cells. Infiltrates are not associated with tubular atrophy. DAB staining at low power (×10) **(a)** and at high power (×40) **(b)**. Kidney transplant biopsy shows minimal interstitial infiltration in the vicinity of atrophic tubules, which is not indicative of rejection. No interstitial infiltrates are seen here compared to native kidney tissue [periodic acid–Schiff (*PAS*) staining, ×20] **(c–f)**. Small bowel biopsies under immunosuppression with locotypical interstitial lymphocytes with dominance of T cells, partly of cytotoxic subtype (CD8-positive), while CD80 positivity can only be seen in single cells [**(g–j)** at low power, ×10]: **(g)** *HE* staining, **(h)** CD3 DAB staining, **(i)** CD80 DAB staining, and **(j)** CD8 DAB staining. Pancreas biopsy prior to transplantation/start of immunosuppression shows interstitial infiltrates and demonstrates CD80 positivity in single cells. DAB staining at low power (×10) **(k)** and at high power (×40) **(l)**.

Immunohistochemistry was done on 2- to 3-μm sections (Leica Bond system) with 3,3-diaminobenzidine (DAB) and periodic acid–Schiff (PAS) staining was performed on 1-μm-thick formalin-fixed, paraffin-embedded sections of kidney, pancreas, and small bowel biopsies of the patient. Kidney biopsies have been taken before and after renal transplantation (RTx) (Figures 2a–f), small bowel biopsy was taken after (Figures 2g–j), and pancreas biopsy before RTx (Figures 2k,l).

### Case Presentation

The, at present, 26-year-old male patient is the first son of consanguine Turkish parents with an unremarkable family history. The initial complaint was persistent diarrhea at the age of 15 months. Vaccinations yielded no titers against pneumococcus, tetanus, and hepatitis. Titers against rubeola, rubella, and parotitis epidemica were markedly reduced. Further immunological workup showed low numbers of B cells and low IgG, but normal IgM levels.

Chronic kidney disease (serum creatinine of 1.1 mg/dl) was first noted during the assessment of his chronic diarrhea at the age of 6 years. He had crossed the third length percentile at 5 years. Despite growth hormone substitution from the age of 5–12 years, the patient remained growth-retarded (final body height, 133 cm; SD = 6.69) (Figure 1B). Detailed workup showed no proteinuria, hypertension, or edema. At the age of 10 years, he suffered from recurrent episodes of pancreatitis. Biopsy revealed chronic pancreatitis with lympho-plasmacellular infiltration. A renal biopsy was performed at the age of 11 years and showed interstitial nephritis. Chronic kidney disease (CKD) rapidly progressed to end-stage renal disease, requiring dialysis at the age of 12 years. After 12 months on hemodialysis, he received a renal allograft from a deceased donor. Immunosuppression consisted of prednisolone, mycophenolate mofetil (MMF), and tacrolimus. Twelve months after RTx post-transplant, lymphoproliferative disease (PTLD) occurred in the cervical lymph nodes. Tacrolimus was stopped, six cycles of rituximab were administered within 8 weeks, and Sirolimus was started in combination with prednisolone and MMF. After this treatment, B cells remained undetectable.

Relapsing infections of both lungs were treated with antibiotics, antivirals, and antifungal agents in the following years. Twenty-four months after RTx, at the age of 14 years, transbronchial lung biopsy revealed bronchiolitis obliterans organizing pneumonia (BOOP). The clinical course was characterized by chronic dry cough and shortness of breath. Histopathologically BOOP was diagnosed by tissue granulation in the bronchiolar lumen, interstitial infiltration with mononuclear cells, and foamy macrophages. Thorax computer tomography showed areas of ground-glass opaque consolidation, swelling, and widening of the bronchial tubes (Figure 1C). The immunosuppressive regimen was changed to prednisolone, MMF, and tacrolimus because of concerns that BOOP may had been induced by Sirolimus. An interstitial cellular rejection was constantly present on four successive kidney graft biopsies and resistant to all immunosuppressive medications. The patient returned to hemodialysis 3 years after transplantation due to graft failure. Finally, WES identified a homozygous stop mutation (p.Q1010^*^) in the *LRBA* gene and established a clear diagnosis at the age of 23 years (Figure 1A).

## Discussion

In LRBA deficiency, decreased IgG antibody production, deficient T cell activation and proliferation, increased apoptosis, and decreased autophagy in B lymphocytes have been reported (5). Charbonnier et al. have also shown alterations on Tregs, increased apoptosis, and a decreased expression of the Treg markers, such as CD25 and cytotoxic T lymphocyte-associated protein 4 (6). These reports point out that LRBA is important for the defense against infections, regulation of cell proliferation, cell death, and immune reactions like hypersensitivity reactions in many tissues due to the dearth of Tregs (6). The native renal pathology in our patient with lympho-histiocytic interstitial infiltration, focal tubular atrophy, and interstitial fibrosis (Figures 2a,b) strikingly resembles the characteristic histology observed in bowel biopsies from LRBA cases (6). In other autoimmune conditions such as the syndrome of tubulointerstitial nephritis and uveitis (TINU), rheumatoid arthritis, and vasculitis tubulointerstitial nephritis are the prototypic forms of renal affection (7, 8).

Although ESRD has not been associated with LRBA deficiency before, renal disease has been previously reported. Among the nine patients responsive to therapy with Abatacept reported by Lo et al. (4). is a 12-year-old child with renal insufficiency. Schreiner et al. reported two Libyan siblings with LRBA deficiency and type 1 diabetes. The authors observed increased serum creatinine and cystatin C levels in the 12-year-old girl, while her younger 5-year-old brother showed no renal involvement (9).

In particular, after kidney transplantation, various complications were misinterpreted as therapy-related or cryptic in our patient. From a present-day perspective, the development of BOOP and likely the PTLD, as well as the previously described clinical features of pancytopenia, the chronic diarrhea, and the pancreatitis resulting in failure to thrive, has to be interpreted within the context of LRBA deficiency. A diagnosis of LRBA deficiency has therapeutic implications. Therapeutic options include the inhibition of lysosome degradation with chloroquine (to prevent CTLA4 loss). However, a trial with 200 mg hydroxychloroquine daily over 6 months had no beneficial impact on the diarrhea, nor did it improve the general condition of our patient. Abatacept, a CTL4 immunoglobulin, binds to the CD80 and CD86 of antigen-presenting cells (APCs) and inhibits co-stimulation of the T cell by an APC. Abatacept mimics the function of the cellular CTLA4 pool (diminished in LRBA deficiency) as a T cell modulator. Abatacept negatively regulates the immune responses by blockading or capturing CD80/CD86 molecules found on antigen-presenting cells (4). In a case series reported by Lo et al., interstitial lung disease, autoimmunity/inflammation, and chronic diarrhea markedly improved under therapy with Abatacept (4) but there are no prospective clinical trials that prove the efficacy and safety of this drug. In our patients' native kidney biopsies, CD80 positivity was detected only in single lymphocytes (Figures 2a,b). Due to the lack of clinical studies, Abatacept was not given to our patient, so far.

Renal replacement therapy, in particular kidney transplantation, has improved the quality of life of many children suffering from isolated or complex renal disease. There are many examples showing that graft survival can be excellent in those children, even in the absence of a clear (molecular) diagnosis. However, there are metabolic diseases and immunodeficiency states in which knowing the underlying disease is indispensable in order to limit or avoid disease-specific complications and institute targeted therapeutic options.

In conclusion, we recommend that WES or an equivalent comprehensive genetic screening should be strongly considered in all patients prior to kidney transplantation, if a clear diagnosis has not been established by other means. We also recommend that LRBA deficiency should be considered in patients presenting with CKD, enteropathy, and symptoms of immunodeficiency. Renal disease may represent an under-recognized feature of the LRBA phenotypic spectrum or long-term sequelae of LRBA deficiency.

## Data Availability Statement

The datasets used and analyzed during the current study are available from the corresponding author on reasonable request.

## Ethics Statement

All investigations were conducted in accordance with the principles of the Declaration of Helsinki and after obtaining written informed consent. Clinical, histopathology, and biochemical data were collected retrospectively from medical charts.

## Consent For Publication

Consent for publication was obtained from the patient before starting the publication process. The pictures shown were previously presented to the patient and are published with his consent.

## Author Contributions

AW, FE, and BB performed the genetic analyses. CT, HG, and LW collected and evaluated clinical, imaging, and histological data. CT, LW, and BB conceived and wrote the manuscript. All authors critically reviewed the manuscript.

### Conflict of Interest

The authors declare that the research was conducted in the absence of any commercial or financial relationships that could be construed as a potential conflict of interest.

## Footnotes

^1^Available online at: https://varbank.ccg.uni-koeln.de
